# Therapeutic strategies of drug repositioning targeting autophagy to induce cancer cell death: from pathophysiology to treatment

**DOI:** 10.1186/s13045-017-0436-9

**Published:** 2017-03-09

**Authors:** Go J. Yoshida

**Affiliations:** 1 Department of Pathological Cell Biology, Medical Research Institute, Tokyo Medical and Dental University, 1-5-45 Yushima, Bunkyo-ku, Tokyo, 113-8510 Japan; 2 Japan Society for the Promotion of Science, 5-3-1 Kojimachi, Chiyoda-ku, Tokyo, 102-0083 Japan

**Keywords:** AMPK, Apoptosis, Autophagic cell death, Cancer stem-like cells, Drug re-positioning, Ferroptosis, mTOR signaling, Nrf2, p53, p62/SQSTM1

## Abstract

The 2016 Nobel Prize in Physiology or Medicine was awarded to the researcher that discovered autophagy, which is an evolutionally conserved catabolic process which degrades cytoplasmic constituents and organelles in the lysosome. Autophagy plays a crucial role in both normal tissue homeostasis and tumor development and is necessary for cancer cells to adapt efficiently to an unfavorable tumor microenvironment characterized by hypo-nutrient conditions. This protein degradation process leads to amino acid recycling, which provides sufficient amino acid substrates for cellular survival and proliferation. Autophagy is constitutively activated in cancer cells due to the deregulation of PI3K/Akt/mTOR signaling pathway, which enables them to adapt to hypo-nutrient microenvironment and exhibit the robust proliferation at the pre-metastatic niche. That is why just the activation of autophagy with mTOR inhibitor often fails in vain. In contrast, disturbance of autophagy–lysosome flux leads to endoplasmic reticulum (ER) stress and an unfolded protein response (UPR), which finally leads to increased apoptotic cell death in the tumor tissue. Accumulating evidence suggests that autophagy has a close relationship with programmed cell death, while uncontrolled autophagy itself often induces autophagic cell death in tumor cells. Autophagic cell death was originally defined as cell death accompanied by large-scale autophagic vacuolization of the cytoplasm. However, autophagy is a “double-edged sword” for cancer cells as it can either promote or suppress the survival and proliferation in the tumor microenvironment. Furthermore, several studies of drug re-positioning suggest that “conventional” agents used to treat diseases other than cancer can have antitumor therapeutic effects by activating/suppressing autophagy. Because of ever increasing failure rates and high cost associated with anticancer drug development, this therapeutic development strategy has attracted increasing attention because the safety profiles of these medicines are well known. Antimalarial agents such as artemisinin and disease-modifying antirheumatic drug (DMARD) are the typical examples of drug re-positioning which affect the autophagy regulation for the therapeutic use. This review article focuses on recent advances in some of the novel therapeutic strategies that target autophagy with a view to treating/preventing malignant neoplasms.

## Background

Cellular degradation processes mainly fall into two categories: macroautophagy (commonly referred to as autophagy) and the ubiquitin-proteasome system. Autophagy is an evolutionarily conserved catabolic process involving the formation of autophagosome vacuoles that engulf cellular macromolecules and dysregulated organelles, leading to their breakdown after fusion with lysosomes [1, 2]. In contrast, ubiquitin-proteasome pathway is composed of two distinct and successive steps: marking the substrate protein with the covalent attachment of numerous ubiquitin molecules and the subsequent degradation of the tagged substrate in the 26S proteasome. Studies have highlighted significant differences between these two degradation systems [3–5]. First, autophagy generates energy efficiently via ATP synthesis in the mitochondria and maintains endoplasmic reticulum (ER) stress mediated by selective degradation of non-functional macromolecules and organelles. In contrast, the proteasome system consumes ATP. Second, the size of the protein targets that can be hydrolyzed via autophagy is virtually unlimited.

Autophagy is driven by mitochondrial depolarization, nutrient starvation, aggregation of toxic proteins, and pathogen infection [6–13]. Thus, viruses, large protein aggregates, and entire organelles are selectively broken down by the autolysosome, which is formed by the fusion of autophagosomes with lysosomes. Autophagy–lysosome flux mainly comprises three steps: (i) formation of phagophores, derived by the ER, mitochondria, and Golgi apparatus [14–16]; (ii) formation of autophagosomes containing macromolecules, pathogenic protein aggregates, and dysregulated mitochondria; and (iii) fusion of autophagosomes with lysosomes to form autolysosomes (with a low pH for protein degradation) [17, 18]. Autophagy is responsible for the clearance of old and dysfunctional organelle. In particular, mitophagy is the selective autophagy-dependent degradation of dysfunctional mitochondria under hypoxic conditions [19]. Mitophagy serves as an adaptive metabolic response that prevents accumulation of high levels of reactive oxygen species (ROS) by removing old/damaged mitochondria [19–21]. Mitochondrial permeability transition is thought to be responsible for the mitophagy of depolarized mitochondria, thereby generating cytotoxic ROS [19, 22].

**Fig. 1:**
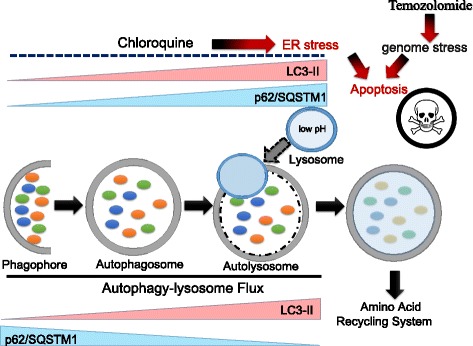
ER stress caused by disruption of autophagy–lysosome flux or conventional chemotherapy confers synergistic therapeutic effects. While p62/SQSTM1 is downregulated during autophagy–lysosome flux, lipidated form of LC-3 (LC-3II) accumulates (*lower panel*). Obstruction of autophagy flux can be pharmacologically induced by chloroquine, which results in ubiquitination, p62 activation, and LC3-II accumulation (*upper panel*). Impairment of the autophagy–lysosome pathway induces apoptosis mainly via excessive ER stress. On the other hand, TMZ is an alkylating agent that induces formation of O^6^-methylguanine in DNA, which in turn induces mismatch pair with thymine during the following cycle of DNA replication. Thus, chloroquine and TMZ exhibit the synergistic therapeutic effect for cancer cells

Mammalian target of rapamycin complex 1 (mTORC1) integrates nutrient and growth factor signaling to promote anabolic metabolisms, such as protein synthesis and lipid synthesis, and to inhibit catabolic pathways, such as lysosome biogenesis and autophagy [23]. Whereas phosphatidylinositol 3-kinase (PI3K)/Akt/mTOR pathway is constitutively activated in numerous kinds of tumors, suppression of PI3K/Akt survival signaling pathway due to the hypo-nutrient microenvironment leads to autophagy induction in tumor cells [24, 25]. During autophagy, the adaptor protein p62/SQSTM1 is consumed, and LC-3 conversion is promoted [26, 27] (lower panel in Fig. 1). Obstruction of autophagy flux can be induced artificially by chloroquine and bafilomycin A1, both of which result in increased levels of ubiquitination, p62 activation, and LC3-II accumulation (upper panel in Fig. 1). The smooth autophagy–lysosome pathway, which is termed autophagy flux, can be disturbed by bafilomycin A1, a specific inhibitor of vacuolar-type H^+^-ATPase. In the presence of bafilomycin A1, autophagosomes fail to exhibit the fusion with lysosomes, leading to the accumulation of numerous immature autophagosomes [3, 28, 29]. Thus, levels of the adaptor protein p62/SQSTM1 and the lipidated mature form of LC3 (LC3-II) increase in the presence of bafilomycin A1 and/or chloroquine under steady state or starvation conditions.

Impairment of the autophagy–lysosome pathway induces apoptosis mainly via excessive ER stress [30, 31]. In contrast, temozolomide (TMZ) is an alkylating agent that induces the formation of O^6^-methylguanine in DNA, which in turn induces mismatch pair with thymine during the following cycle of DNA replication [32, 33]. Accumulating evidence strongly suggests the role of O^6^-methylguanine-DNA methyltransferase (MGMT) expression in the acquired resistance to TMZ in malignant glioma and acute leukemia cells [34, 35]. The mechanisms underlying the action of TMZ and the pathways by which glioma cells escape death have yet to be adequately elucidated; however, genome stress due to TMZ synergistically induces apoptosis in collaboration with accumulated ER stress upon chloroquine treatment (Fig. 1) [2, 3].

### Activation of autophagy in tumor tissues

Cancer cells tend to activate autophagy constitutively via metabolic reprogramming [36–38]. For a typical instance, tumor cells activate AMP-activated protein kinase (AMPK), a key energy sensor that regulates cellular metabolism, to maintain energy homeostasis [38, 39]. Activated AMPK regulates the autophagy-dependent amino acid recycling system in collaboration with FIP200 and ULK1. Also, phosphorylation of AMPK suppresses mTORC1 mediated inactivation of Raptor or activation of TSC2 [40, 41]. Under conditions of nutrient sufficiency, increased mTOR activity prevents ULK1 activation by phosphorylating ULK1 on Ser 757, thereby disrupting the interaction between ULK1 and AMPK [8].

In addition, several molecular machineries have been proposed to explain the tumor suppressive function of autophagy: (i) accumulation of p62, a substrate of autophagy, leads to NF-κB activation [42]; (ii) accumulation of p62 stabilizes the transcription factor nuclear factor erythroid 2-related factor 2 (Nrf2), which imparts tumor cells with resistance to hypoxic stress [43]; (iii) retention of damaged organelles, including mitochondria [44]. These mechanisms may be cell- and stimulus-type specific.

Constitutive activation of autophagy in tumor tissue is a challenge regarding therapeutic resistance [45, 46]. Activated autophagy protects glioblastoma multiforme (GBM) cells from the hyper-oxidative, hypoxic, and hypo-nutrient tumor microenvironment [47, 48]. For example, TMZ, an alkylating agent used to treat GBM and anaplastic astrocytoma [49, 50], induces autophagy and subsequent therapeutic resistance, which is why Nrf2 inhibitors exhibit a therapeutic effect when used in combination with TMZ [48, 51]. Indeed, Nrf2 knockdown enhances autophagy induced by TMZ, which attenuates the cancer proliferation [51]. Furthermore, the flavonoid chrysin, which is a potent Nrf2 inhibitor, has been shown to effectively overcome the drug resistance by downregulating the PI3K/Akt and ERK pathways [52].

**Fig. 2:**
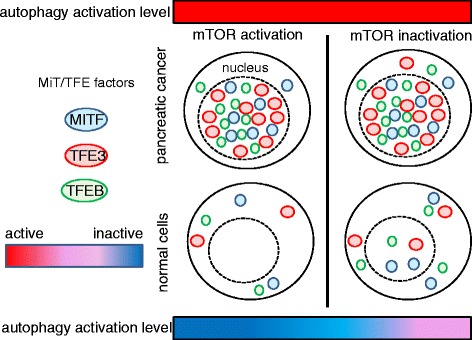
Nuclear translocation of MiT/TFE protein is responsible for the constitutive activation of autophagy–lysosome pathway in cancer cells. Compared with normal cells, greater amounts of MiT/TFE transcriptional factors (i.e., MITF, TFE3, and TFEB) accumulate in the nuclei of cancer cells under nutrient-insufficient conditions. These transcriptional factors drive expression of genes related to autophagylysosome flux. Surprisingly, even under mTOR-inactivated conditions (such as starvation), cancer cells express high levels of Mit/TFE proteins in the nucleus, which may explain the constitutive activation of autophagy independent of mTOR signaling. Note that the *red bar* indicates the enhanced autophagic activation, while the *blue bar* indicates the suppressed autophagic regulation

Notably, recent investigations indicate the importance of transcriptional factors for regulating constitutive activation of autophagy [53, 54]. Pancreatic adenocarcinoma cells exhibit constitutive nuclear expression of TFE3 and MITF despite displaying suppressed mTORC1 signaling in the presence of Torin1, which is an mTOR inhibitor. Constitutive activation of transcriptional factors TFE3 and MITF in the nuclei of pancreatic cancer cells is critical for autophagy–lysosome function [53]. These basic helix–loop–helix–leucine zipper (bHLH-Zip) transcriptional factors are well known to be involved in the differentiation of osteoclasts, mast cells, and melanocytes [55], and exhibit the translocation in renal cell carcinoma [56]. Thus, the persistent nuclear localization of MiT/TFE factors regardless of mTORC1 signal modification induces and maintains the robust activation of anabolic pathways in tumor cells, while cancer cells survive and proliferate owing to the fine-tuning metabolic regulation and the adaptation to metabolic stress afforded by activation of autophagy and lysosome-dependent protein degradation. Activated mTORC1 phosphorylates MiT/TFE proteins, a process that inhibits nuclear translocation mediated by importin 8, which itself belongs to the importin-β family of nucleocytoplasmic transporters (Fig. 2) [57]. Also, depletion of c-Myc impairs autophagy flux, thereby reducing phosphorylation of JNK1 and its downstream target anti-apoptotic molecule Bcl2. Knockdown of this proto-oncogenic transcriptional factor disrupts autophagosome formation [58]. The functional relevance of this observation reinforces the attractiveness of targeting Myc as a therapeutic strategy for cancer [59–61], since autophagy promotes cell survival under the stress conditions (i.e., nutrient depletion and hypoxia) often encountered by established tumor cells.

### Intra-tumoral heterogeneity and metabolic stress responses

As previously explained, cancer cells highly depend on autophagy in the tumor microenvironment [45, 46]. Differences in the mechanism(s) of autophagy activation determine metabolic symbiosis, which explains the heterogeneous therapeutic response to anticancer therapies targeting tumor metabolism [38, 62]. For a typical example, there is metabolic cross-talk between cancer stem cells (CSCs) and non-CSCs and between cancer cells and cancer-associated fibroblasts (CAFs) [38, 63, 64]. CSCs exist at the top of the hierarchical tumor cell society and possess several biological features including resistance to oxidative stress and chronic inflammation [65–67], capacity for rapid repair of damaged DNA [68, 69], ability to adapt to an unfavorable tumor microenvironment lacking of glucose or growth factors [70, 71], plasticity between the proliferative and quiescent cell cycle conditions [72, 73], and importantly, metabolic reprogramming [38, 74–76]. Furthermore, CAFs in the tumor stroma exhibit robust activity in terms of aerobic glycolysis and autophagy due to loss of caveolin 1 (Cav-1) expression [63, 77]. Cav-1 is a scaffold protein involved in several cellular processes, including cholesterol homeostasis, vesicular transport, and regulation of signal transduction. Although Cav-1 functions as a tumor suppressor molecule in several solid cancers [78–80], its loss from stromal cells positive for α-smooth muscle actin (α-SMA) leads to robust proliferation, increased extracellular matrix production, and activated TGF-β signaling [81]. Also, a metabolic hallmark of a constitutive myofibroblastic phenotype is achieving higher levels of aerobic glycolysis and autophagy than in neighboring cancer cells [63]. CAFs depend on autophagy; importantly, activation of autophagy in the tumor stroma leads to chemo-resistance [63, 82].

Notably, circulating tumor cells express high levels of epithelial cell adhesion molecule (EpCAM), also known as CD147 [83, 84]. EpCAM interacts with three major amino acid transporters: monocarboxylate transporters (MCTs), SLC1A5 (also known as ASCT2), and SLC7A5 (also known as LAT1) [71, 85]. Given that LAT1 transports branched side-chain amino acids such as l-leucine into cells in exchange for efflux of intracellular amino acids such as l-glutamine, EpCAM-induced stabilization of LAT1 distribution at the cellular membrane negatively regulates the mTOR signaling pathway [71, 86]. EpCAM is a marker of functional CSCs and, as such, regulates metabolic stress [38, 71]. EpCAM expression is, at least in part, responsible for the observed heterogeneity of tumor cell metabolism [87–89]. CSCs expressing high levels of EpCAM can adapt to the hypo-nutrient tumor microenvironment better than non-CSCs that express low levels of EpCAM. Taken together, the different degrees of autophagy activation in CSCs, non-CSCs, and CAFs lead to heterogeneity and cross-talk between pathways responsible for tumor metabolism.

**Fig. 3:**
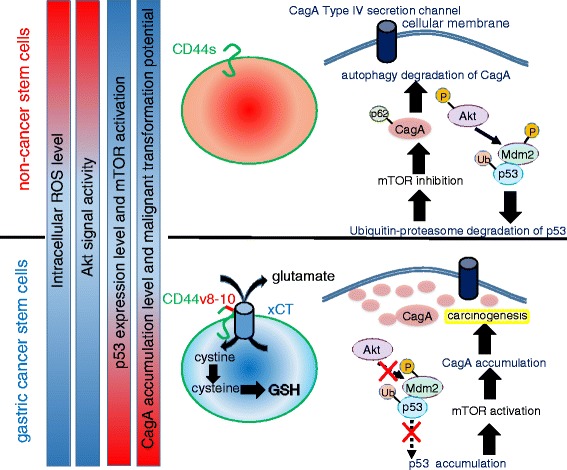
CD44 variant-xCT axis-mediated ROS regulation determines the malignant transformation of gastric epithelial cells showing CagA accumulation. Stabilization of xCT (cystine/glutamate antiporter) at the cell membrane in gastric epithelial stem cells due to high CD44v8-10 expression promotes glutathione synthesis, thereby inactivating the Akt signaling pathway. Phosphorylated Akt in CD44v-negative cells induces ubiquitin-proteasome-dependent degradation of p53 in the cytoplasm. Activated Akt signal transduction in non-cancer stem-like cells expressing the standard isoform of CD44 exhibit selective autophagy-mediated degradation of CagA. CagA is translocated from *H. pylori* via type IV secretion channels, and importantly, accumulation of this pathogenic protein in CD44v-expressing cancer stem-like cells leads to carcinogenesis and maintenance of “stemness.” Note that the *red bar* shows the relatively high level, while the *blue bar* indicates the low level

Of note, the autophagy-dependent and selective degradation of cytotoxin-associated gene A (CagA), the type IV secretion effector of *Helicobacter pylori* (*H. pylori*), is activated by reduced levels of intracellular glutathione (GSH), resulting in redox stress and activation of Akt [12]. Gastric CSCs expressing high levels of a CD44 variant containing exons 8–10 (CD44v8-10) are resistant to ROS due to the robust GSH synthesis mediated by stabilization of xCT (a cysteine/glutamate antiporter) at the cellular membrane (Fig. 3) [65, 90, 91]. Gastric cancer cells expressing high levels of CD44v8-10 fail to support the autophagy-dependent degradation of CagA. Mounting evidence has demonstrated that the accumulation of intracellular CagA due to autophagy inhibition is observed in CSCs derived from human gastric adenocarcinoma [12, 92, 93]. Taken together, these studies suggest that both of the ubiquitin-proteasome pathway and the selective autophagy machinery contribute to the emergence of gastric cancer-initiating cells derived from tissue stem cells expressing CD44v8-10.

### Therapeutic strategies associated with drug re-positioning

Several reports show that certain drugs conventionally used to treat non-malignant diseases exhibit anticancer effects, a phenomenon referred to as oncology drug re-positioning [94, 95]. Biopharmaceutical manufacturers who attempt to increase productivity through novel discovery technologies have fallen short of achieving the desired results when attempting to develop de novo anticancer drugs. Given the costs associated with the discovery, development, registration, and commercialization of new therapeutic agents, it has been increasingly difficult for pharmaceutical companies to achieve an adequate return on investment for difficult-to-cure diseases. Re-positioning conventional drugs may rectify this. Thus, an increasing number of pharmaceutical companies are aggressively scanning the existing pharmacopoeia for re-positioning candidates. For instance, chloroquine is used to treat malaria and autoimmune disorders such as systemic lupus erythematosus and rheumatoid arthritis. However, chloroquine also blocks autolysosome formation by disrupting fusion between mature autophagosomes and lysosomes; this agent acts synergistically with TMZ to induce GRP78/BiP-dependent ER stress accumulation (Fig. 1) [1, 96–98]. Compared with novel molecular-targeting drugs, conventional agents are not only pharmacologically safe but are also cheaper than specialized drugs. Combination therapy with multiple signal transduction inhibitors has been tested in a clinical setting with the aim of preventing tumor cells from activating alternative survival and proliferation signaling pathways and acquiring resistance to a single targeting agent [99–101]; however, this therapeutic strategy has unrealistically high costs. In contrast, the use of conventional medications would be much cheaper.

### Therapeutic strategies that promote synergistic antitumor effects

Some conventional agents show synergistic antitumor effects when used alongside chemotherapeutic agents or radiation [95, 102, 103]. For instance, pyrimethamine (Pyr) is an orally administered drug used to treat infections caused by protozoan parasites such as malaria. Pyr is an anti-folate agent that blocks dihydrofolate reductase (DHFR) [104]. DHFR inhibitors have been studied as anticancer drugs because they are selectively toxic to rapidly proliferating cells [104, 105]. Tommasino et al. recently investigated the therapeutic effect of novel derivatives of Pyr, including iso-pyrimethamine (Iso-Pyr), *m*-nitropyrimethamine (MNP), methylbenzoprim (MBP), and *m*-azidopyrimethamine ethanesulfonate salt (MZPES), against metastatic melanoma cells both in vitro and in vivo [95]. Among these derivatives, MBP induces cell cycle arrest and apoptosis upon cathepsin B activation. Cathepsin B is a lysosomal cysteine protease that directs cells toward both autophagy and apoptosis [106, 107]. It is notable that the in vivo concentration of MBP necessary for a significant therapeutic effect is approximately fivefold lower than that of Pyr [95], which strongly suggests that the antitumor effects of MBP may be as efficient as those of other anti-folate drugs already in use (e.g., methotrexate) but with less severe adverse effects.

Furthermore, high-throughput screening identifies F-AMP as combination agents for gastrointestinal stromal tumor (GIST) therapy with imatinib mesylate. The majority cases of GIST which are resistant to conventional chemotherapy and radiation have been well controlled with imatinib mesylate. However, these effects are often short-lived, because some of the cases with GIST demonstrate a median time to progression of approximately 2 years [108–110]. F-AMP inhibits DNA synthesis by interfering with ribonucleotide reductase and DNA polymerase [103, 111]. Given that clinical cases of advanced GIST progressing on tyrosine kinase therapy frequently have secondary mutations, there is a rationale for testing combination therapies that target receptor tyrosine kinase-independent pathways, such as those that block DNA synthesis and lead to enhanced DNA damage [103]. Thus, synergistic antitumor effect prevents the emergence of secondary mutations which are responsible for the acquired resistance.

### Drug re-positioning for the treatment of solid tumors

Several investigations have revealed the therapeutic usefulness of drug re-positioning for the treatment of solid tumors [112–114]. It has been recently identified, for example, that the novel therapeutic strategy targeting autophagy in GBM focuses not only on conventional drugs but also on endogenous neurotransmitters in the synapse. A recent study described the chemical screening of 680 neurochemical compounds using patient-derived GBM neural stem cells (GNS) and the subsequent identification of dopamine receptor D4 (DRD4) antagonists as selective inhibitors of GNS growth and inducers of normal neural stem cell differentiation and LC-3 puncta formation [114]. Xenograft experiments revealed that a DRD4 antagonist acted synergistically with TMZ to activate autophagy and inhibit GNS proliferation. CSCs tend to exhibit autophagy activation either under steady conditions or upon exposure to various stresses; this results in robust survival and proliferation within the niche. Importantly, when autophagy flux is obstructed, the amounts of both p62/SQSTM1 (an adaptor protein for selective autophagy) and LC3-II (the lipidated mature form of LC-3) increase. This leads to ER stress and, ultimately, apoptosis. DRD4 antagonists suppress the PDGF receptor-β/ERK1/2 (p44/p42-MAPK) signaling axis and disrupt autophagy flux in GBM stem cells, leading to apoptosis via caspase-3-mediated cleavage of poly (ADP-ribose) polymerase (PARP) [114]. It is notable that fananserin, a drug that acts as a potent antagonist of the serotonin 5HT2A receptor and the dopamine D4 receptor, has long been used as a sedative and as a treatment for schizophrenia, bipolar disorder, and antianxiety [115]. Furthermore, the purinergic receptor P2Y_12_ inhibitor ticlopidine, which is an anticoagulant drug used to prevent transient ischemic attacks and stroke, increases intracellular cAMP levels in low-grade glioma and high-grade astrocytoma and promotes autophagy flux (Table 1). Notably, tricyclic antidepressants such as imipramine act synergistically with ticlopidine to promote autophagy in glioma cells by further increasing intracellular cAMP concentrations [116]. Taken together, these studies suggest that induction of excessive autophagy in cancer cells using conventional drugs leads to autophagic cell death.

**Table 1:** Typical examples of drug re-positioning targeting autophagy in cancer cells

Name of the agent (the type of the drug)	Conventional application	Mechanism of action to exhibit the antitumor effect	Targeting tumor types	References
Sulfasalazine (cystine/glutamate antiporter inhibitor)	Ulcerative colitis, rheumatoid arthritis	To decrease GSH synthesis by the disruption of cystine uptake via xCT transporter and, therefore, enhancing ROS leads to ferroptosis, which is the autophagic cell death due to the excessive degradation of ferritin.	Gastric cancer, breast cancer, head and neck squamous carcinoma, non-small cell lung cancer	[65, 131, 135–139]
Chloroquine (antimalarial drug)	Autoimmune diseases such as lupus and rheumatoid arthritis	To disrupt the fusion of autophagosomes with lysosomes (the formation of autolysosomes) and to enhance GRP78/BiP-dependent ER stress. Remarkably, TMZ and chloroquine show the synergistic therapeutic effect.	Colon cancer, malignant melanoma, hepatocellular carcinoma, low-grade glioma, high-grade astrocytomas	[96]
Fananserin (dopamine receptor 4 antagonist)	Schizophrenia, bipolar disorder, antianxiety and sedative effects	To suppress PDGFR-β/ERK signal pathway, to induce G_0_/G_1_ cell cycle arrest, and to disrupt autophagy–lysosome pathway in which enough ER stress accumulates for apoptosis of glioma cells to occur	High-grade astrocytomas (anaplastic astrocytoma grade III and glioblastoma multiforme)	[114, 115]
Ticlopidine (purinergic receptor P2Y12 inhibitor)	Anticoagulant drug to prevent transient ischemic attack (TIA) and stroke	To increase intracellular cAMP level and promote autophagy flux. Notably, tricyclic antidepressants such as imipramine promote autophagy in glioma cells synergistically with this drug by further elevating intracellular cAMP concentration.	Low-grade glioma, high-grade astrocytomas	[116]
Valproic acid (a short-chain fatty acid HDAC inhibitor)	Epilepsy such as tonic-clonic seizures	To upregulate CDKN1A/B and downregulate c-Myc, thereby augmenting mTOR inhibitor to induce autophagic cell death	Cutaneous T cell lymphoma, Burkitt leukemia/lymphoma	[126, 148]
Terfenadine (histamine receptor H_1_ antagonist)	Autoimmune diseases such as allergic dermatitis	To induce ROS-mediated DNA damage, autophagy, and apoptosis independent of p53 via the attenuated secretion of VEGF in hypoxic area	Malignant melanoma	[140–142]

### Drug re-positioning of antimalarial agents and the underlying molecular machinery

Beclin 1 (the homolog of yeast autophagy-related gene 6 (Atg6)) was the first mammalian autophagy protein to be identified [117]. Beclin 1 comprises a class III PI3K complex that plays a role in autophagosome formation [118, 119]. Also, haploinsufficiency of Beclin 1, which is directly phosphorylated by AMPK, increases the incidence of spontaneous tumor development in Beclin 1^+/−^ heterozygous mutant mice [120]. Tumor types included lung adenocarcinomas, hepatocellular tumors, and lymphomas showing Nrf2 accumulation and p62-positive inclusion bodies. Although autophagy tends to suppress tumor initiation, it increases invasive and metastatic potential. This is the “double-edged sword” of autophagy with respect to malignant neoplasms [121, 122]. Dihydroartemisinin (DHA) is a critical inducer of c-Jun NH2-terminal kinase (JNK)-mediated Beclin 1 expression in pancreatic cancer cells [123]. Treatment of human pancreatic cancer cell lines with DHA activates caspase-3 and induces conversion of LC-3 to its lipidated form, hallmarks of apoptosis and autophagy, respectively [123]. Both transient small interfering RNA (si-RNA)-mediated depletion of Beclin 1 and pharmacological suppression of class III PI3K by 3-methyladenine (3-MA) lead to reduced numbers of double-membrane vacuoles (called autophagosomes) within cells. Thus, Beclin1 plays a fundamental role in DHA-induced activation of autophagy. Furthermore, DHA causes ROS-induced JNK phosphorylation in a concentration- and time-dependent manner [123]. JNK activation is responsible for Bcl-2 phosphorylation, which increases autophagy by disrupting the competitive interaction between Beclin 1 and Bcl-2 [124]. Bcl-2 regulates autophagy by directly binding to Beclin 1, which partially explains the relationship between autophagy and apoptosis. Although Jia et al. did not mention this specifically [123], it is highly likely that DHA induces autophagic cancer cell death, defined as cell death due to excessive autophagy. Surprisingly enough, autophagic cell death in mouse embryonic fibroblasts (MEFs) established from Bax/Bak double-knockout mice, which are resistant to apoptotic cell death, was rescued by 3-MA treatment [44, 125]. Notably, given that JNK activation is observed during autophagic cell death, it may be that DHA induces this type of cell death as well as apoptosis [126]. Thus, JNK inhibitors can rescue autophagic cell death in a reversible manner.

### Drug re-positioning of DMARDs and the underlying molecular machinery

Artemisinin, which is known to be an antimalarial agent [127, 128], induces iron-dependent necrotic cell death, also referred to as ferroptosis, in cancer cells [129, 130]. Ferroptosis is recognized in various human diseases, including ischemic tissue damage and malignancy. Recent research revealed the close relationship between ferroptosis and autophagic cell death. Pharmacological induction of ferroptosis leads to excessive activation of selective autophagy, which in turn results in the degradation of ferritin and the ferritinophagy cargo receptor NCOA4 [131]. In addition, because ferroptosis is triggered by excessive ROS levels due to insufficient amounts of GSH, system X_C_^−^ is likely to be involved. System X_C_^−^ is an amino acid antiporter that typically mediates the exchange of extracellular l-cysteine (l-Cys2) and intracellular l-glutamate (l-Glu) across the cellular plasma membrane. It is composed of a light chain, xCT, and a heavy chain, 4F2 heavy chain (4F2hc); thus, it belongs to the family of heterodimeric amino acid transporters [132, 133]. Sulfasalazine, which is a disease-modifying antirheumatic drug (DMARD), has long been used to treat rheumatoid arthritis and ulcerative colitis. DMARDs are a group of medications commonly used in patients with autoimmunue disorders characterized by rheumatoid arthritis. Some of these drugs are also used in treating other conditions such as ankylosing spondylitis, psoriatic arthritis, and systemic lupus erythematosus. DMARDs are mainly composed of methotrexate, d-penicillamine, and sulfasalazine [134]. Notably, sulfasalazine inhibits the cysteine/glutamate antiporter, thereby attenuating GSH synthesis by disrupting cysteine uptake via system X_C_^−^ [65, 135, 136]. This DMARD is also an effective treatment for glioma-associated brain edema due to increased intracellular concentrations of glutamate [137, 138]. Increased ROS levels lead to ferroptosis, a form of autophagic cell death caused by excessive degradation of ferritin and NCOA4 [131]. Remarkably, a clinical trial of combination treatment with chemotherapy with sulfasalazine has been performed in patients with non-small-cell lung cancer and patients with advanced gastric tumors without driver gene mutations such as *RAS* (G12V) (Table 1) [91, 139]. Taken together, these studies suggest that chemical or drug screening should be undertaken to identify the novel antitumor therapeutic effects of drug re-positioning in a clinical setting.

### Drug re-positioning and molecular mechanisms associated with p53 and epigenetics

The histamine receptor H_1_ antagonist terfenadine, which is used to treat patients with autoimmune diseases such as allergic dermatitis, suppresses invasion and metastasis of malignant melanoma cells. Terfenadine induces ROS-mediated DNA damage, autophagy, and p53-independent apoptosis by attenuating secretion of vascular endothelial growth factor in hypoxic areas [140]. Activation of p53 increases mitochondrial membrane permeabilization, cytochrome *c* release, and caspase-9 activation. ROS inhibition by vitamin E partially attenuates induction of p73 and Noxa expression, but not that of p53 and p21. This strongly suggests that Noxa expression and apoptotic cell death are regulated independently of p53. In malignant melanoma cells, a strong apoptotic stimulus conferred by terfenadine triggers Ca^2+^-dependent DNA damage and activation of caspase-2 as the predominant mechanisms which induce apoptosis via the mitochondrial pathway [141]. Caspase-2, which is activated by an autoproteolytic mechanism in response to DNA damage, interacts directly with mitochondria to trigger mitochondrial membrane permeabilization and cytochrome *c* release [141, 142].

Indeed, recent studies show that excessive induction of autophagy in aggressively proliferating cancer cells is an essential therapeutic target of histone deacetylase (HDAC) inhibitors [126, 143, 144]. HDAC inhibitor-induced autophagy is mainly caused by transcriptional activation of FOXO1, which promotes autophagy via mTOR signal suppression and ATGs upregulation [144]. Remarkably enough, while hyper-acetylation of ATGs has been implicated in starvation-induced autophagy, deacetylation of proteins crucial for autophagy including ATG5, ATG7, ATG12, and LC3 is implicated in autophagy induction by starvation [144, 145]. Given that mTOR signaling, which is aberrantly activated in lymphoma, plays a major role in tumor cell growth [146, 147]; Dong et al. demonstrated that HDAC inhibitors and mTOR inhibitors work synergistically to inhibit Burkitt B cell lymphomas showing constitutive activation of PI3K/Akt signaling and c-Myc overexpression [126]. In the clinical settings, valproic acid (VPA; a short-chain fatty acid HDAC inhibitor) is widely used as an anticonvulsant; however, it also exhibits antitumor activity [148]. In lymphoma cells, HDAC inhibition by VPA is essential for the autophagy-enhancing effects observed when it is used in combination with the mTOR inhibitor temsirolimus [126]. Therefore, epigenetic modulation via VPA inhibition is a promising method of inducing autophagic cell death in malignant neoplasms. Still, much remains to be elucidated about the relationship between HDAC-mediated epigenetic regulation and autophagy induction or suppression.

### Drug re-positioning of natural and functional food ingredients

**Fig. 4:**
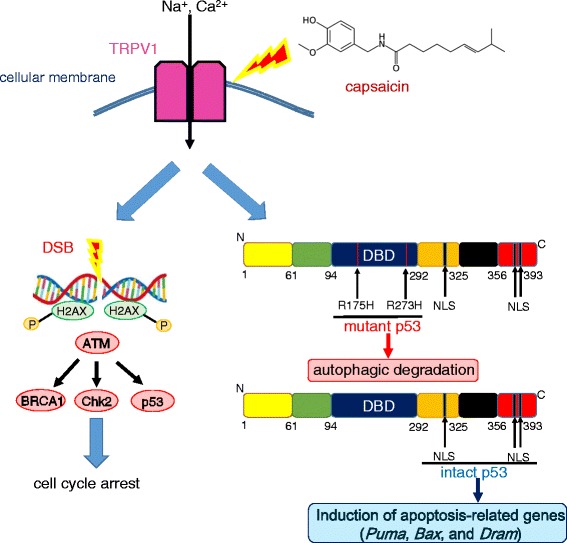
Capsaicin induces simultaneous autophagic degradation of mutant p53 and reactivation of wild-type p53. Capsaicin activates TRPV1, leading to double-strand DNA breaks and phosphorylation of histone H2AX. ATM kinase phosphorylates and activates a number of DNA repair and checkpoint proteins, including p53, Brca1, and Chk2, ultimately causing cell cycle arrest. On the other hand, capsaicin induces autophagic degradation of p53R175H and p53R273H and reactivates intact p53 that does not harbor mutations in the DNA-binding domain. Thus, expression of apoptotic genes such as *Puma*, *Bax*, and *DRAM* increases

Common ingredients of many foods can also be subject to drug re-positioning. For example, capsaicin (*trans*-8-methyl-*N*-vanillyl-6-nonenamide), the major pungent ingredient in “hot” chili peppers, elicits a sensation of burning by selectively activating sensory neurons that convey peripheral information about noxious stimuli to the central nervous system [149]. Capsaicin binds to a receptor called transient receptor potential cation channel subfamily V member 1 (TRPV1), the archetypal member of the vanilloid TRP family [150]. TRPV1 functions as the mediator of chemical and physical stimuli at nociceptor peripheral terminals and plays a crucial role in thermal inflammatory hyperalgesia. Garufi et al. recently investigated the antitumor effects of capsaicin, which occur via autophagy-mediated specific degradation of a p53 mutant [151]. It is widely accepted that tumor-associated p53 mutations such as p53R175H and p53R273H, rather than the heterozygous loss of wild-type tumor-suppressing p53, cause the malignant phenotype [152, 153]. Numerous mutant p53 proteins acquire oncogenic properties that enable cancer cells to increase their capacity for invasion, colonization, and proliferation within the pre-metastatic niche [91, 154]. Remarkably, Garufi et al. revealed that capsaicin-induced reactivation of p53 increases the susceptibility of mutant p53-harboring tumor cells to conventional anticancer agents such as ADR and CDDP [151]. In the presence of capsaicin, TRPV1 activation leads to double-strand breaks and phosphorylation of histone H2AX [155, 156]. Ataxia-telangiectasia (A-T)-mutated (ATM) kinase functions by phosphorylating and activating some DNA repair and checkpoint proteins, including p53, H2AX, 53BP1, Brca1, and Chk2, which ultimately induce cell cycle arrest [157, 158]. Furthermore, reactivated wild-type p53 induces expression of apoptotic genes such as *Puma*, *Bax*, and *DRAM* (damage-regulated autophagy modulator). In particular, DRAM, which is induced only by a few natural compounds, is upregulated by genotoxic stress. DRAM is required for p53-induced autophagy and apoptosis [159, 160]. p53-mediated cell death in response to cellular stress requires both DRAM-induced autophagy and other pro-death signals (mediated by targets such as PUMA, NOXA, and Bax) to elicit a full death response (Fig. 4).

Sulforaphane (SFN) is produced by hydrolysis of glucoraphanin after ingestion of cruciferous vegetables, particularly broccoli and broccoli sprouts [161]. SFN acts as a tumor-preventive molecule by activating Nrf2 [162]. Nrf2 binds to Kelch-like ECH-associated protein 1 (KEAP1) in the cytoplasm under steady-state conditions; however, Nrf2 dissociates from KEAP1 and translocates to the nucleus upon exposure to redox stress [163, 164]. Activation of the antioxidant response element is dependent on Nrf2 and induces expression of heme oxygenase 1 (HO-1), NAD(P)H-quinone oxidoreductase (NQO1), GST, superoxide dismutase 3 (SOD3), and glucuronosyltransferase-1a6 (UGT-1a6). These enzymes have cytoprotective, antioxidant, and anti-inflammatory effects. SFN also induces autophagy in human breast cancer cells, a process inhibited by bafilomycin A1 but not by 3-MA [165]. This suggests that SFN does not disrupt the formation of the autophagosome, but rather that of the autolysosome, the structure formed after fusion of the autophagosome with the lysosome [165, 166]. SFN-induced autophagy increases susceptibility to apoptosis by modulating Bax, BCL-2, caspase-3, PARP-1, and the mitochondrial membrane potential [165, 167]. The cross-talk between signals that activate autophagy and apoptosis requires further investigation if we are to better understand the therapeutic significance of drug re-positioning in terms of the molecular and signaling machineries.

## Conclusions

Conventional agents are not only pharmacologically safe but also cheaper than specialized anticancer drugs. However, much remains to be discovered in terms of the cross-talk between signals that mediate autophagy and apoptosis [168, 169]. The 2016 Nobel Prize in Physiology or Medicine was awarded to Emeritus Professor Yoshinori Ohsumi (Tokyo Institute of Technology) for his discovery of the autophagy machinery [170]; therefore, improving our understanding of the mechanisms and relationships between conventional drugs, chemotherapy, and autophagy in the clinical setting is an important research topic. Such an approach will enable us to develop novel anticancer treatments that target signal transduction pathways related to cancer cell death.

## Abbreviations

AMPK: AMP-activated protein kinase
ARE: Antioxidant response element
Atg: Autophagy-related gene
ATM: Ataxia-telangiectasia (A-T) mutated
CAF: Cancer-associated fibroblasts
CagA: Cytotoxin-associated gene A
Cav-1: Caveolin 1
CDK1: Cyclin-dependent kinase 1
CSCs: Cancer stem-like cells
DHA: Dihydroartemisinin
DHFR: Dihydrofolate reductase
DRAM: Damage-regulated autophagy modulator
DRD4: Dopamine receptor D4
EpCAM: Epithelial cell adhesion molecule
ER: Endoplasmic reticulum
GBM: Glioblastoma multiforme
GIST: Gastrointestinal stromal tumor
GNS: GBM neural stem cells
GSH: Glutathione
*H. pylori*: *Helicobacter pylori*
HDAC: Histone deacetylases
HO-1: Heme oxygenase 1
IL: Interleukin
Iso-Pyr: Iso-pyrimethamine
JNK: c-Jun NH2-terminal kinase
KEAP1: Kelch-like ECH-associated protein 1
MBP: Methylbenzoprim
MCT: Monocarboxylate transporter
MEFs: Mouse embryonic fibroblasts
MNP: *m*-Nitropyrimethamine
MPT: Mitochondrial permeability transition
mTOR: Mammalian target of rapamycin
MZPES: *m*-Azidopyrimethamine ethanesulfonate salt
NQO-1: NAD(P)H-quinone oxidoreductase-1
Nrf2: Nuclear factor erythroid 2-related factor 2
PARP: Poly (ADP-ribose) polymerase
PI3K: Phosphatidylinositol 3-kinase
Pyr: Pyrimethamine
ROS: Reactive oxygen species
SLE: Systemic lupus erythematosus
TEM: Transmission electron microscope
TMZ: Temozolomide
TRPV1: Transient receptor potential cation channel subfamily V member 1
UPR: Unfolded protein response
VEGF: Vascular endothelial growth factor
VPA: Valproic acid

## References

[1] Glick D, Barth S, Macleod KF (2010). Autophagy: cellular and molecular mechanisms. J Pathol.

[2] Das G, Shravage BV, Baehrecke EH. Regulation and function of autophagy during cell survival and cell death. Cold Spring Harb Perspect Biol. 2012;4(6). 10.1101/cshperspect.a008813 PMC3367545 22661635

[3] Kaur J, Debnath J (2015). Autophagy at the crossroads of catabolism and anabolism. Nat Rev Mol Cell Biol.

[4] Korolchuk VI, Menzies FM, Rubinsztein DC (2010). Mechanisms of cross-talk between the ubiquitin-proteasome and autophagy-lysosome systems. FEBS Lett.

[5] Ciechanover A (2005). Intracellular protein degradation: from a vague idea thru the lysosome and the ubiquitin-proteasome system and onto human diseases and drug targeting. Cell Death Differ.

[6] Wang Y, Nartiss Y, Steipe B, McQuibban GA, Kim PK (2012). ROS-induced mitochondrial depolarization initiates PARK2/PARKIN-dependent mitochondrial degradation by autophagy. Autophagy.

[7] Lee J, Giordano S, Zhang J (2012). Autophagy, mitochondria and oxidative stress: cross-talk and redox signalling. Biochem J.

[8] Kim J, Kundu M, Viollet B, Guan KL (2011). AMPK and mTOR regulate autophagy through direct phosphorylation of Ulk1. Nat Cell Biol.

[9] Mihaylova MM, Shaw RJ (2011). The AMPK signalling pathway coordinates cell growth, autophagy and metabolism. Nat Cell Biol.

[10] Metcalf DJ, Garcia-Arencibia M, Hochfeld WE, Rubinsztein DC (2012). Autophagy and misfolded proteins in neurodegeneration. Exp Neurol.

[11] Jimenez-Sanchez M, Thomson F, Zavodszky E, Rubinsztein DC (2012). Autophagy and polyglutamine diseases. Prog Neurobiol.

[12] Tsugawa H, Suzuki H, Saya H, Hatakeyama M, Hirayama T, Hirata K, Nagano O, Matsuzaki J, Hibi T (2012). Reactive oxygen species-induced autophagic degradation of Helicobacter pylori CagA is specifically suppressed in cancer stem-like cells. Cell Host Microbe.

[13] Wileman T (2013). Autophagy as a defence against intracellular pathogens. Essays Biochem.

[14] Chan SN, Tang BL (2013). Location and membrane sources for autophagosome formation—from ER-mitochondria contact sites to Golgi-endosome-derived carriers. Mol Membr Biol.

[15] Hamasaki M, Furuta N, Matsuda A, Nezu A, Yamamoto A, Fujita N, Oomori H, Noda T, Haraguchi T, Hiraoka Y (2013). Autophagosomes form at ER-mitochondria contact sites. Nature.

[16] Marchi S, Patergnani S, Pinton P (2014). The endoplasmic reticulum-mitochondria connection: one touch, multiple functions. Biochim Biophys Acta.

[17] Mauvezin C, Neisch AL, Ayala CI, Kim J, Beltrame A, Braden CR, Gardner MK, Hays TS, Neufeld TP (2016). Coordination of autophagosome-lysosome fusion and transport by a Klp98A-Rab14 complex in Drosophila. J Cell Sci.

[18] Yu L, McPhee CK, Zheng L, Mardones GA, Rong Y, Peng J, Mi N, Zhao Y, Liu Z, Wan F (2010). Termination of autophagy and reformation of lysosomes regulated by mTOR. Nature.

[19] Ding WX, Yin XM (2012). Mitophagy: mechanisms, pathophysiological roles, and analysis. Biol Chem.

[20] Saito T, Sadoshima J (2015). Molecular mechanisms of mitochondrial autophagy/mitophagy in the heart. Circ Res.

[21] Zhang H, Bosch-Marce M, Shimoda LA, Tan YS, Baek JH, Wesley JB, Gonzalez FJ, Semenza GL (2008). Mitochondrial autophagy is an HIF-1-dependent adaptive metabolic response to hypoxia. J Biol Chem.

[22] Lemasters JJ, Qian T, He L, Kim JS, Elmore SP, Cascio WE, Brenner DA (2002). Role of mitochondrial inner membrane permeabilization in necrotic cell death, apoptosis, and autophagy. Antioxid Redox Signal.

[23] Kim YC, Guan KL (2015). mTOR: a pharmacologic target for autophagy regulation. J Clin Invest.

[24] Porta C, Paglino C, Mosca A (2014). Targeting PI3K/Akt/mTOR signaling in cancer. Front Oncol.

[25] Arcaro A, Guerreiro AS (2007). The phosphoinositide 3-kinase pathway in human cancer: genetic alterations and therapeutic implications. Curr Genomics.

[26] Pankiv S, Clausen TH, Lamark T, Brech A, Bruun JA, Outzen H, Overvatn A, Bjorkoy G, Johansen T (2007). p62/SQSTM1 binds directly to Atg8/LC3 to facilitate degradation of ubiquitinated protein aggregates by autophagy. J Biol Chem.

[27] Klionsky DJ, Abdelmohsen K, Abe A, Abedin MJ, Abeliovich H, Acevedo Arozena A, Adachi H, Adams CM, Adams PD, Adeli K (2016). Guidelines for the use and interpretation of assays for monitoring autophagy (3rd edition). Autophagy.

[28] Loos B, du Toit A, Hofmeyr JH (2014). Defining and measuring autophagosome flux-concept and reality. Autophagy.

[29] Yoshimori T, Yamamoto A, Moriyama Y, Futai M, Tashiro Y (1991). Bafilomycin A1, a specific inhibitor of vacuolar-type H(+)-ATPase, inhibits acidification and protein degradation in lysosomes of cultured cells. J Biol Chem.

[30] Hoyer-Hansen M, Jaattela M (2007). Connecting endoplasmic reticulum stress to autophagy by unfolded protein response and calcium. Cell Death Differ.

[31] Senft D, Ronai ZA (2015). UPR, autophagy, and mitochondria crosstalk underlies the ER stress response. Trends Biochem Sci.

[32] Zhang J, Stevens MF, Bradshaw TD (2012). Temozolomide: mechanisms of action, repair and resistance. Curr Mol Pharmacol.

[33] Stepanenko AA, Andreieva SV, Korets KV, Mykytenko DO, Baklaushev VP, Huleyuk NL, Kovalova OA, Kotsarenko KV, Chekhonin VP, Vassetzky YS (2016). Temozolomide promotes genomic and phenotypic changes in glioblastoma cells. Cancer Cell Int.

[34] Happold C, Roth P, Wick W, Schmidt N, Florea AM, Silginer M, Reifenberger G, Weller M (2012). Distinct molecular mechanisms of acquired resistance to temozolomide in glioblastoma cells. J Neurochem.

[35] Seiter K, Katragadda S, Ponce D, Rasul M, Ahmed N (2009). Temozolomide and cisplatin in relapsed/refractory acute leukemia. J Hematol Oncol.

[36] Phan LM, Yeung SC, Lee MH (2014). Cancer metabolic reprogramming: importance, main features, and potentials for precise targeted anti-cancer therapies. Cancer Biol Med.

[37] Ward PS, Thompson CB (2012). Metabolic reprogramming: a cancer hallmark even warburg did not anticipate. Cancer Cell.

[38] Yoshida GJ (2015). Metabolic reprogramming: the emerging concept and associated therapeutic strategies. J Exp Clin Cancer Res.

[39] Kuhajda FP (2008). AMP-activated protein kinase and human cancer: cancer metabolism revisited. Int J Obes (Lond).

[40] Hardie DG (2007). AMP-activated/SNF1 protein kinases: conserved guardians of cellular energy. Nat Rev Mol Cell Biol.

[41] Hardie DG, Ross FA, Hawley SA (2012). AMPK: a nutrient and energy sensor that maintains energy homeostasis. Nat Rev Mol Cell Biol.

[42] Mathew R, Karp CM, Beaudoin B, Vuong N, Chen G, Chen HY, Bray K, Reddy A, Bhanot G, Gelinas C (2009). Autophagy suppresses tumorigenesis through elimination of p62. Cell.

[43] Takamura A, Komatsu M, Hara T, Sakamoto A, Kishi C, Waguri S, Eishi Y, Hino O, Tanaka K, Mizushima N (2011). Autophagy-deficient mice develop multiple liver tumors. Genes Dev.

[44] Shimizu S, Konishi A, Nishida Y, Mizuta T, Nishina H, Yamamoto A, Tsujimoto Y (2010). Involvement of JNK in the regulation of autophagic cell death. Oncogene.

[45] Sui X, Chen R, Wang Z, Huang Z, Kong N, Zhang M, Han W, Lou F, Yang J, Zhang Q (2013). Autophagy and chemotherapy resistance: a promising therapeutic target for cancer treatment. Cell Death Dis.

[46] Yang ZJ, Chee CE, Huang S, Sinicrope FA (2011). The role of autophagy in cancer: therapeutic implications. Mol Cancer Ther.

[47] Jawhari S, Ratinaud MH, Verdier M (2016). Glioblastoma, hypoxia and autophagy: a survival-prone ‘menage-a-trois. Cell Death Dis.

[48] Yan Y, Xu Z, Dai S, Qian L, Sun L, Gong Z (2016). Targeting autophagy to sensitive glioma to temozolomide treatment. J Exp Clin Cancer Res.

[49] Friedman HS, Kerby T, Calvert H (2000). Temozolomide and treatment of malignant glioma. Clin Cancer Res.

[50] Mason WP, Cairncross JG (2005). Drug insight: temozolomide as a treatment for malignant glioma—impact of a recent trial. Nat Clin Pract Neurol.

[51] Zhou Y, Wang HD, Zhu L, Cong ZX, Li N, Ji XJ, Pan H, Wang JW, Li WC (2013). Knockdown of Nrf2 enhances autophagy induced by temozolomide in U251 human glioma cell line. Oncol Rep.

[52] Gao AM, Ke ZP, Shi F, Sun GC, Chen H (2013). Chrysin enhances sensitivity of BEL-7402/ADM cells to doxorubicin by suppressing PI3K/Akt/Nrf2 and ERK/Nrf2 pathway. Chem Biol Interact.

[53] Perera RM, Stoykova S, Nicolay BN, Ross KN, Fitamant J, Boukhali M, Lengrand J, Deshpande V, Selig MK, Ferrone CR (2015). Transcriptional control of autophagy-lysosome function drives pancreatic cancer metabolism. Nature.

[54] Martina JA, Diab HI, Lishu L, Jeong AL, Patange S, Raben N, Puertollano R (2014). The nutrient-responsive transcription factor TFE3 promotes autophagy, lysosomal biogenesis, and clearance of cellular debris. Sci Signal.

[55] Martina JA, Diab HI, Li H, Puertollano R (2014). Novel roles for the MiTF/TFE family of transcription factors in organelle biogenesis, nutrient sensing, and energy homeostasis. Cell Mol Life Sci.

[56] Kauffman EC, Ricketts CJ, Rais-Bahrami S, Yang Y, Merino MJ, Bottaro DP, Srinivasan R, Linehan WM (2014). Molecular genetics and cellular features of TFE3 and TFEB fusion kidney cancers. Nat Rev Urol.

[57] Raices M, D'Angelo MA (2012). Nuclear pore complex composition: a new regulator of tissue-specific and developmental functions. Nat Rev Mol Cell Biol.

[58] Toh PP, Luo S, Menzies FM, Rasko T, Wanker EE, Rubinsztein DC (2013). Myc inhibition impairs autophagosome formation. Hum Mol Genet.

[59] Granato M, Rizzello C, Romeo MA, Yadav S, Santarelli R, D'Orazi G, Faggioni A, Cirone M (2016). Concomitant reduction of c-Myc expression and PI3K/AKT/mTOR signaling by quercetin induces a strong cytotoxic effect against Burkitt’s lymphoma. Int J Biochem Cell Biol.

[60] Huang H, Weng H, Zhou H, Qu L (2014). Attacking c-Myc: targeted and combined therapies for cancer. Curr Pharm Des.

[61] Prochownik EV, Vogt PK (2010). Therapeutic targeting of Myc. Genes Cancer.

[62] Warmoes MO, Locasale JW (2014). Heterogeneity of glycolysis in cancers and therapeutic opportunities. Biochem Pharmacol.

[63] Pavlides S, Whitaker-Menezes D, Castello-Cros R, Flomenberg N, Witkiewicz AK, Frank PG, Casimiro MC, Wang C, Fortina P, Addya S (2009). The reverse Warburg effect: aerobic glycolysis in cancer associated fibroblasts and the tumor stroma. Cell Cycle.

[64] Zhao H, Yang L, Baddour J, Achreja A, Bernard V, Moss T, Marini JC, Tudawe T, Seviour EG, San Lucas FA (2016). Tumor microenvironment derived exosomes pleiotropically modulate cancer cell metabolism. Elife.

[65] Ishimoto T, Nagano O, Yae T, Tamada M, Motohara T, Oshima H, Oshima M, Ikeda T, Asaba R, Yagi H (2011). CD44 variant regulates redox status in cancer cells by stabilizing the xCT subunit of system xc(−) and thereby promotes tumor growth. Cancer Cell.

[66] Ishimoto T, Oshima H, Oshima M, Kai K, Torii R, Masuko T, Baba H, Saya H, Nagano O (2010). CD44+ slow-cycling tumor cell expansion is triggered by cooperative actions of Wnt and prostaglandin E2 in gastric tumorigenesis. Cancer Sci.

[67] Yoshida GJ (2016). Emerging role of epithelial-mesenchymal transition in hepatic cancer. J Exp Clin Cancer Res.

[68] Maugeri-Sacca M, Bartucci M, De Maria R (2012). DNA damage repair pathways in cancer stem cells. Mol Cancer Ther.

[69] Wang QE (2015). DNA damage responses in cancer stem cells: Implications for cancer therapeutic strategies. World J Biol Chem.

[70] Vlashi E, Lagadec C, Vergnes L, Matsutani T, Masui K, Poulou M, Popescu R, Della Donna L, Evers P, Dekmezian C (2011). Metabolic state of glioma stem cells and nontumorigenic cells. Proc Natl Acad Sci U S A.

[71] Yoshida GJ, Saya H (2014). EpCAM expression in the prostate cancer makes the difference in the response to growth factors. Biochem Biophys Res Commun.

[72] Yoshida GJ. The heterogeneity of cancer stem-like cells at the invasive front. Cancer Cell Int. 2017;17:23. doi:10.1186/s12935-017-0393-y. 10.1186/s12935-017-0393-y PMC5307924 28289330

[73] Weinberg R, Fisher DE, Rich J (2010). Dynamic and transient cancer stem cells nurture melanoma. Nat Med.

[74] Shen YA, Wang CY, Hsieh YT, Chen YJ, Wei YH (2015). Metabolic reprogramming orchestrates cancer stem cell properties in nasopharyngeal carcinoma. Cell Cycle.

[75] Saga I, Shibao S, Okubo J, Osuka S, Kobayashi Y, Yamada S, Fujita S, Urakami K, Kusuhara M, Yoshida K (2014). Integrated analysis identifies different metabolic signatures for tumor-initiating cells in a murine glioblastoma model. Neuro Oncol.

[76] Wu Z, Wei D, Gao W, Xu Y, Hu Z, Ma Z, Gao C, Zhu X, Li Q (2015). TPO-induced metabolic reprogramming drives liver metastasis of colorectal cancer CD110+ tumor-initiating cells. Cell Stem Cell.

[77] Chen D, Che G (2014). Value of caveolin-1 in cancer progression and prognosis: emphasis on cancer-associated fibroblasts, human cancer cells and mechanism of caveolin-1 expression (review). Oncol Lett.

[78] Pinilla SM, Honrado E, Hardisson D, Benitez J, Palacios J (2006). Caveolin-1 expression is associated with a basal-like phenotype in sporadic and hereditary breast cancer. Breast Cancer Res Treat.

[79] Wiechen K, Diatchenko L, Agoulnik A, Scharff KM, Schober H, Arlt K, Zhumabayeva B, Siebert PD, Dietel M, Schafer R (2001). Caveolin-1 is down-regulated in human ovarian carcinoma and acts as a candidate tumor suppressor gene. Am J Pathol.

[80] Zhang ZB, Cai L, Zheng SG, Xiong Y, Dong JH (2009). Overexpression of caveolin-1 in hepatocellular carcinoma with metastasis and worse prognosis: correlation with vascular endothelial growth factor, microvessel density and unpaired artery. Pathol Oncol Res.

[81] Sotgia F, Del Galdo F, Casimiro MC, Bonuccelli G, Mercier I, Whitaker-Menezes D, Daumer KM, Zhou J, Wang C, Katiyar S (2009). Caveolin-1−/− null mammary stromal fibroblasts share characteristics with human breast cancer-associated fibroblasts. Am J Pathol.

[82] Fang WB, Yao M, Cheng N (2014). Priming cancer cells for drug resistance: role of the fibroblast niche. Front Biol (Beijing).

[83] Gorges TM, Tinhofer I, Drosch M, Rose L, Zollner TM, Krahn T, von Ahsen O (2012). Circulating tumour cells escape from EpCAM-based detection due to epithelial-to-mesenchymal transition. BMC Cancer.

[84] Grover PK, Cummins AG, Price TJ, Roberts-Thomson IC, Hardingham JE (2014). Circulating tumour cells: the evolving concept and the inadequacy of their enrichment by EpCAM-based methodology for basic and clinical cancer research. Ann Oncol.

[85] Xu D, Hemler ME (2005). Metabolic activation-related CD147-CD98 complex. Mol Cell Proteomics.

[86] Wang Q, Holst J (2015). L-type amino acid transport and cancer: targeting the mTORC1 pathway to inhibit neoplasia. Am J Cancer Res.

[87] Hensley CT, Faubert B, Yuan Q, Lev-Cohain N, Jin E, Kim J, Jiang L, Ko B, Skelton R, Loudat L (2016). Metabolic heterogeneity in human lung tumors. Cell.

[88] Robertson-Tessi M, Gillies RJ, Gatenby RA, Anderson AR (2015). Impact of metabolic heterogeneity on tumor growth, invasion, and treatment outcomes. Cancer Res.

[89] Sengupta D, Pratx G (2016). Imaging metabolic heterogeneity in cancer. Mol Cancer.

[90] Yoshida GJ, Saya H (2014). Inversed relationship between CD44 variant and c-Myc due to oxidative stress-induced canonical Wnt activation. Biochem Biophys Res Commun.

[91] Yoshida GJ, Saya H (2016). Therapeutic strategies targeting cancer stem cells. Cancer Sci.

[92] Hatakeyama M (2006). The role of Helicobacter pylori CagA in gastric carcinogenesis. Int J Hematol.

[93] Yong X, Tang B, Li BS, Xie R, Hu CJ, Luo G, Qin Y, Dong H, Yang SM (2015). Helicobacter pylori virulence factor CagA promotes tumorigenesis of gastric cancer via multiple signaling pathways. Cell Commun Signal.

[94] Langedijk J, Mantel-Teeuwisse AK, Slijkerman DS, Schutjens MH (2015). Drug repositioning and repurposing: terminology and definitions in literature. Drug Discov Today.

[95] Tommasino C, Gambardella L, Buoncervello M, Griffin RJ, Golding BT, Alberton M, Macchia D, Spada M, Cerbelli B, d'Amati G (2016). New derivatives of the antimalarial drug Pyrimethamine in the control of melanoma tumor growth: an in vitro and in vivo study. J Exp Clin Cancer Res.

[96] Kimura T, Takabatake Y, Takahashi A, Isaka Y (2013). Chloroquine in cancer therapy: a double-edged sword of autophagy. Cancer Res.

[97] Li J, Lee AS (2006). Stress induction of GRP78/BiP and its role in cancer. Curr Mol Med.

[98] Lee AS (2007). GRP78 induction in cancer: therapeutic and prognostic implications. Cancer Res.

[99] Patel TA, Dave B, Rodriguez AA, Chang JC, Perez EA, Colon-Otero G (2014). Dual HER2 blockade: preclinical and clinical data. Breast Cancer Res.

[100] Puri N, Salgia R (2008). Synergism of EGFR and c-Met pathways, cross-talk and inhibition, in non-small cell lung cancer. J Carcinog.

[101] Wen W, Wu J, Liu L, Tian Y, Buettner R, Hsieh MY, Horne D, Dellinger TH, Han ES, Jove R (2015). Synergistic anti-tumor effect of combined inhibition of EGFR and JAK/STAT3 pathways in human ovarian cancer. Mol Cancer.

[102] Pasquier E, Andre N, Street J, Chougule A, Rekhi B, Ghosh J, Philip DS, Meurer M, MacKenzie KL, Kavallaris M (2016). Effective management of advanced angiosarcoma by the synergistic combination of propranolol and vinblastine-based metronomic chemotherapy: a bench to bedside study. EBioMedicine.

[103] Pessetto ZY, Ma Y, Hirst JJ, von Mehren M, Weir SJ, Godwin AK (2014). Drug repurposing identifies a synergistic combination therapy with imatinib mesylate for gastrointestinal stromal tumor. Mol Cancer Ther.

[104] Schweitzer BI, Dicker AP, Bertino JR (1990). Dihydrofolate reductase as a therapeutic target. FASEB J.

[105] Gangjee A, Kurup S, Namjoshi O (2007). Dihydrofolate reductase as a target for chemotherapy in parasites. Curr Pharm Des.

[106] de Castro MA, Bunt G, Wouters FS (2016). Cathepsin B launches an apoptotic exit effort upon cell death-associated disruption of lysosomes. Cell Death Discov.

[107] Zhou J, Tan SH, Nicolas V, Bauvy C, Yang ND, Zhang J, Xue Y, Codogno P, Shen HM (2013). Activation of lysosomal function in the course of autophagy via mTORC1 suppression and autophagosome-lysosome fusion. Cell Res.

[108] Rink L, Skorobogatko Y, Kossenkov AV, Belinsky MG, Pajak T, Heinrich MC, Blanke CD, von Mehren M, Ochs MF, Eisenberg B (2009). Gene expression signatures and response to imatinib mesylate in gastrointestinal stromal tumor. Mol Cancer Ther.

[109] Tarn C, Merkel E, Canutescu AA, Shen W, Skorobogatko Y, Heslin MJ, Eisenberg B, Birbe R, Patchefsky A, Dunbrack R (2005). Analysis of KIT mutations in sporadic and familial gastrointestinal stromal tumors: therapeutic implications through protein modeling. Clin Cancer Res.

[110] Wang CM, Huang K, Zhou Y, Du CY, Ye YW, Fu H, Zhou XY, Shi YQ (2010). Molecular mechanisms of secondary imatinib resistance in patients with gastrointestinal stromal tumors. J Cancer Res Clin Oncol.

[111] Puccio CA, Mittelman A, Lichtman SM, Silver RT, Budman DR, Ahmed T, Feldman EJ, Coleman M, Arnold PM, Arlin ZA (1991). A loading dose/continuous infusion schedule of fludarabine phosphate in chronic lymphocytic leukemia. J Clin Oncol.

[112] Shim JS, Liu JO (2014). Recent advances in drug repositioning for the discovery of new anticancer drugs. Int J Biol Sci.

[113] Bernstein WB, Dennis PA (2008). Repositioning HIV protease inhibitors as cancer therapeutics. Curr Opin HIV AIDS.

[114] Dolma S, Selvadurai HJ, Lan X, Lee L, Kushida M, Voisin V, Whetstone H, So M, Aviv T, Park N (2016). Inhibition of dopamine receptor D4 impedes autophagic flux, proliferation, and survival of glioblastoma stem cells. Cancer Cell.

[115] Heuillet E, Petitet F, Mignani S, Malleron JL, Lavayre J, Neliat G, Doble A, Blanchard JC (1996). The naphtosultam derivative RP 62203 (fananserin) has high affinity for the dopamine D4 receptor. Eur J Pharmacol.

[116] Shchors K, Massaras A, Hanahan D (2015). Dual targeting of the autophagic regulatory circuitry in gliomas with repurposed drugs elicits cell-lethal autophagy and therapeutic benefit. Cancer Cell.

[117] Kametaka S, Okano T, Ohsumi M, Ohsumi Y (1998). Apg14p and Apg6/Vps30p form a protein complex essential for autophagy in the yeast, Saccharomyces cerevisiae. J Biol Chem.

[118] Cao Y, Klionsky DJ (2007). Physiological functions of Atg6/Beclin 1: a unique autophagy-related protein. Cell Res.

[119] Liang XH, Kleeman LK, Jiang HH, Gordon G, Goldman JE, Berry G, Herman B, Levine B (1998). Protection against fatal Sindbis virus encephalitis by beclin, a novel Bcl-2-interacting protein. J Virol.

[120] Qu X, Yu J, Bhagat G, Furuya N, Hibshoosh H, Troxel A, Rosen J, Eskelinen EL, Mizushima N, Ohsumi Y (2003). Promotion of tumorigenesis by heterozygous disruption of the beclin 1 autophagy gene. J Clin Invest.

[121] Thorburn A (2014). Autophagy and its effects: making sense of double-edged swords. PLoS Biol.

[122] White E, DiPaola RS (2009). The double-edged sword of autophagy modulation in cancer. Clin Cancer Res.

[123] Jia G, Kong R, Ma ZB, Han B, Wang YW, Pan SH, Li YH, Sun B (2014). The activation of c-Jun NH(2)-terminal kinase is required for dihydroartemisinin-induced autophagy in pancreatic cancer cells. J Exp Clin Cancer Res.

[124] Wei Y, Sinha S, Levine B (2008). Dual role of JNK1-mediated phosphorylation of Bcl-2 in autophagy and apoptosis regulation. Autophagy.

[125] Shimizu S, Yoshida T, Tsujioka M, Arakawa S (2014). Autophagic cell death and cancer. Int J Mol Sci.

[126] Dong LH, Cheng S, Zheng Z, Wang L, Shen Y, Shen ZX, Chen SJ, Zhao WL (2013). Histone deacetylase inhibitor potentiated the ability of MTOR inhibitor to induce autophagic cell death in Burkitt leukemia/lymphoma. J Hematol Oncol.

[127] Gatto F, Nielsen J (2016). Systematic analysis of overall survival and interactions between tumor mutations and drug treatment. J Hematol Oncol.

[128] Ashley EA, Dhorda M, Fairhurst RM, Amaratunga C, Lim P, Suon S, Sreng S, Anderson JM, Mao S, Sam B (2014). Spread of artemisinin resistance in Plasmodium falciparum malaria. N Engl J Med.

[129] Dixon SJ, Lemberg KM, Lamprecht MR, Skouta R, Zaitsev EM, Gleason CE, Patel DN, Bauer AJ, Cantley AM, Yang WS (2012). Ferroptosis: an iron-dependent form of nonapoptotic cell death. Cell.

[130] Vanden Berghe T, Linkermann A, Jouan-Lanhouet S, Walczak H, Vandenabeele P (2014). Regulated necrosis: the expanding network of non-apoptotic cell death pathways. Nat Rev Mol Cell Biol.

[131] Gao M, Monian P, Pan Q, Zhang W, Xiang J, Jiang X (2016). Ferroptosis is an autophagic cell death process. Cell Res.

[132] Bridges RJ, Natale NR, Patel SA (2012). System xc(−) cystine/glutamate antiporter: an update on molecular pharmacology and roles within the CNS. Br J Pharmacol.

[133] Lewerenz J, Hewett SJ, Huang Y, Lambros M, Gout PW, Kalivas PW, Massie A, Smolders I, Methner A, Pergande M (2013). The cystine/glutamate antiporter system x(c)(-) in health and disease: from molecular mechanisms to novel therapeutic opportunities. Antioxid Redox Signal.

[134] Dale J, Alcorn N, Capell H, Madhok R (2007). Combination therapy for rheumatoid arthritis: methotrexate and sulfasalazine together or with other DMARDs. Nat Clin Pract Rheumatol.

[135] Dai L, Cao Y, Chen Y, Parsons C, Qin Z (2014). Targeting xCT, a cystine-glutamate transporter induces apoptosis and tumor regression for KSHV/HIV-associated lymphoma. J Hematol Oncol.

[136] Yoshikawa M, Tsuchihashi K, Ishimoto T, Yae T, Motohara T, Sugihara E, Onishi N, Masuko T, Yoshizawa K, Kawashiri S (2013). xCT inhibition depletes CD44v-expressing tumor cells that are resistant to EGFR-targeted therapy in head and neck squamous cell carcinoma. Cancer Res.

[137] Buckingham SC, Campbell SL, Haas BR, Montana V, Robel S, Ogunrinu T, Sontheimer H (2011). Glutamate release by primary brain tumors induces epileptic activity. Nat Med.

[138] Sehm T, Fan Z, Ghoochani A, Rauh M, Engelhorn T, Minakaki G, Dorfler A, Klucken J, Buchfelder M, Eyupoglu IY (2016). Sulfasalazine impacts on ferroptotic cell death and alleviates the tumor microenvironment and glioma-induced brain edema. Oncotarget.

[139] Shitara K, Doi T, Nagano O, Imamura CK, Ozeki T, Ishii Y, Tsuchihashi K, Takahashi S, Nakajima TE, Hironaka S (2017). Dose-escalation study for the targeting of CD44v+ cancer stem cells by sulfasalazine in patients with advanced gastric cancer (EPOC1205). Gastric Cancer.

[140] Jeong HJ, Oh HA, Nam SY, Han NR, Kim YS, Kim JH, Lee SJ, Kim MH, Moon PD, Kim HM (2013). The critical role of mast cell-derived hypoxia-inducible factor-1alpha in human and mice melanoma growth. Int J Cancer.

[141] Jangi SM, Ruiz-Larrea MB, Nicolau-Galmes F, Andollo N, Arroyo-Berdugo Y, Ortega-Martinez I, Diaz-Perez JL, Boyano MD (2008). Terfenadine-induced apoptosis in human melanoma cells is mediated through Ca2+ homeostasis modulation and tyrosine kinase activity, independently of H1 histamine receptors. Carcinogenesis.

[142] Nicolau-Galmes F, Asumendi A, Alonso-Tejerina E, Perez-Yarza G, Jangi SM, Gardeazabal J, Arroyo-Berdugo Y, Careaga JM, Diaz-Ramon JL, Apraiz A (2011). Terfenadine induces apoptosis and autophagy in melanoma cells through ROS-dependent and -independent mechanisms. Apoptosis.

[143] Gammoh N, Lam D, Puente C, Ganley I, Marks PA, Jiang X (2012). Role of autophagy in histone deacetylase inhibitor-induced apoptotic and nonapoptotic cell death. Proc Natl Acad Sci U S A.

[144] Zhang J, Ng S, Wang J, Zhou J, Tan SH, Yang N, Lin Q, Xia D, Shen HM (2015). Histone deacetylase inhibitors induce autophagy through FOXO1-dependent pathways. Autophagy.

[145] Banreti A, Sass M, Graba Y (2013). The emerging role of acetylation in the regulation of autophagy. Autophagy.

[146] Westin JR (2014). Status of PI3K/Akt/mTOR pathway inhibitors in lymphoma. Clin Lymphoma Myeloma Leuk.

[147] Zoncu R, Efeyan A, Sabatini DM (2011). mTOR: from growth signal integration to cancer, diabetes and ageing. Nat Rev Mol Cell Biol.

[148] Cang S, Ma Y, Liu D (2009). New clinical developments in histone deacetylase inhibitors for epigenetic therapy of cancer. J Hematol Oncol.

[149] Maggi CA, Meli A (1988). The sensory-efferent function of capsaicin-sensitive sensory neurons. Gen Pharmacol.

[150] Pingle SC, Matta JA, Ahern GP (2007). Capsaicin receptor: TRPV1 a promiscuous TRP channel. Handb Exp Pharmacol.

[151] Garufi A, Pistritto G, Cirone M, D'Orazi G (2016). Reactivation of mutant p53 by capsaicin, the major constituent of peppers. J Exp Clin Cancer Res.

[152] Muller PA, Vousden KH (2013). p53 mutations in cancer. Nat Cell Biol.

[153] Olivier M, Hollstein M, Hainaut P (2010). TP53 mutations in human cancers: origins, consequences, and clinical use. Cold Spring Harb Perspect Biol.

[154] Bar J, Moskovits N, Oren M (2010). Involvement of stromal p53 in tumor-stroma interactions. Semin Cell Dev Biol.

[155] Amantini C, Ballarini P, Caprodossi S, Nabissi M, Morelli MB, Lucciarini R, Cardarelli MA, Mammana G, Santoni G (2009). Triggering of transient receptor potential vanilloid type 1 (TRPV1) by capsaicin induces Fas/CD95-mediated apoptosis of urothelial cancer cells in an ATM-dependent manner. Carcinogenesis.

[156] Masumoto K, Tsukimoto M, Kojima S (2013). Role of TRPM2 and TRPV1 cation channels in cellular responses to radiation-induced DNA damage. Biochim Biophys Acta.

[157] Abraham RT (2001). Cell cycle checkpoint signaling through the ATM and ATR kinases. Genes Dev.

[158] Bolderson E, Richard DJ, Zhou BB, Khanna KK (2009). Recent advances in cancer therapy targeting proteins involved in DNA double-strand break repair. Clin Cancer Res.

[159] Crighton D, Wilkinson S, O'Prey J, Syed N, Smith P, Harrison PR, Gasco M, Garrone O, Crook T, Ryan KM (2006). DRAM, a p53-induced modulator of autophagy, is critical for apoptosis. Cell.

[160] Crighton D, Wilkinson S, Ryan KM (2007). DRAM links autophagy to p53 and programmed cell death. Autophagy.

[161] Nakagawa K, Umeda T, Higuchi O, Tsuzuki T, Suzuki T, Miyazawa T (2006). Evaporative light-scattering analysis of sulforaphane in broccoli samples: quality of broccoli products regarding sulforaphane contents. J Agric Food Chem.

[162] Myzak MC, Dashwood RH (2006). Chemoprotection by sulforaphane: keep one eye beyond Keap1. Cancer Lett.

[163] Kansanen E, Kuosmanen SM, Leinonen H, Levonen AL (2013). The Keap1-Nrf2 pathway: mechanisms of activation and dysregulation in cancer. Redox Biol.

[164] Taguchi K, Motohashi H, Yamamoto M (2011). Molecular mechanisms of the Keap1-Nrf2 pathway in stress response and cancer evolution. Genes Cells.

[165] Kanematsu S, Uehara N, Miki H, Yoshizawa K, Kawanaka A, Yuri T, Tsubura A (2010). Autophagy inhibition enhances sulforaphane-induced apoptosis in human breast cancer cells. Anticancer Res.

[166] Mizushima N, Yoshimori T, Levine B (2010). Methods in mammalian autophagy research. Cell.

[167] Pawlik A, Wiczk A, Kaczynska A, Antosiewicz J, Herman-Antosiewicz A (2013). Sulforaphane inhibits growth of phenotypically different breast cancer cells. Eur J Nutr.

[168] El-Khattouti A, Selimovic D, Haikel Y, Hassan M (2013). Crosstalk between apoptosis and autophagy: molecular mechanisms and therapeutic strategies in cancer. J Cell Death.

[169] Maiuri MC, Zalckvar E, Kimchi A, Kroemer G (2007). Self-eating and self-killing: crosstalk between autophagy and apoptosis. Nat Rev Mol Cell Biol.

[170] Van Noorden R, Ledford H (2016). Medicine nobel for research on how cells ‘eat themselves’. Nature.

